# Structured sampling of molecularly classified mossy fiber inputs by cerebellar granule cells

**DOI:** 10.3389/fncom.2026.1717379

**Published:** 2026-06-22

**Authors:** Xiaomeng Han, Elif Sevde Meral, Jeff W. Lichtman

**Affiliations:** 1 Department of Molecular and Cellular Biology, Harvard University, Cambridge, MA, United States; 2 Bezmialem Vakif University School of Medicine, Istanbul, Türkiye

**Keywords:** cerebellum, computational modeling, connectomics, correlated light and electron microscopy (CLEM), granule cell, mossy fiber terminal, synaptic input selection, VGLuT1

## Abstract

The cerebellar granule cell layer receives mossy fiber inputs from diverse brain regions, yet the principles governing how individual granule cells sample distinct types of inputs remain poorly understood. Using a volumetric correlated light and electron microscopy (vCLEM) dataset from an adult female mouse cerebellum, in which VGluT1-positive and VGluT1-negative mossy fiber terminals are molecularly distinguished, we reconstructed granule cell and mossy fiber connectivity to examine input selection rules. To test whether connectivity can be explained by spatial proximity alone, we developed distance-limited random sampling null models based on empirical cell spatial arrangement, simulating adult and developmental sampling regimes. Granule cell-centered analyses showed that granule cells shared less innervation from the same mossy fiber than expected by chance, indicating that structured sampling cannot be explained by distance-constrained random connectivity alone. Moreover, subpopulations of granule cells preferentially sample either VGluT1-positive or VGluT1-negative mossy fibers. In contrast, mossy fiber–centered analysis showed that individual terminals distributed their outputs across granule cells in a pattern broadly consistent with random sampling. However, sampling in the adult model was more selective than in the model that reflects developmental processes. Together, our findings demonstrated structured, non-random sampling of cerebellar VGluT1-positive and VGluT1-negative mossy fiber inputs and provide insight into how granule cells integrate molecularly distinct inputs to support cerebellar computation.

## Introduction

The cerebellar granule cell layer is the most densely packed neuronal structure in the brain and serves as the first major site of synaptic integration in the cerebellar cortex (Voogd and Glickstein, 1998; Eccles et al., 1967). Granule cells receive inputs from extrinsic mossy fibers–axons originating from diverse brain regions such as the pontine nuclei, vestibular nuclei, reticular formation, and the spinal cord–that convey sensory, motor, and state-related information (Voogd and Glickstein, 1998; Apps and Hawkes, 2009). Intrinsic mossy fiber terminals originate from unipolar brush cells (UBCs) in the vestibulocerebellum (Nunzi and Mugnaini, 2000). The convergence of these heterogeneous inputs allows granule cells to expand and transform incoming signals, forming the basis for cerebellar computations, including timing, prediction, and learning (Marr, 1969; Albus, 1971; Chabrol et al., 2015; Billings et al., 2014).

Based on the Marr-Albus theory (Marr, 1969; Albus, 1971), granule cells sample mossy fibers in a random, unstructured manner to enhance pattern separation. Recent advances in connectomics (Swanson and Lichtman, 2016; Helmstaedter, 2013) using volumetric electron microscopy (vEM) (Peddie et al., 2022) have enabled detailed reconstruction of neural circuits. Using these approaches, granule cell input sampling has been shown to be not entirely random: individual granule cells share mossy fiber inputs more frequently than expected under anatomically constrained random models, and mossy fiber terminals are represented unevenly across granule cells (Nguyen et al., 2022). These findings are consistent with theoretical predictions that sparse and structured synaptic connectivity can optimize pattern separation and the dimensionality of cerebellum-like circuits, including granule cell networks (Litwin-Kumar et al., 2017; Cayco-Gajic et al., 2017). Despite the well-characterized anatomical organization of this layer, the principles by which individual granule cells sample from different mossy fiber types remain largely unknown.

Mossy fibers differ not only in their anatomical origin but also in their molecular identity. Expression of vesicular glutamate transporters (VGluTs) (Fremeau et al., 2004) distinguishes mossy fiber classes: many precerebellar neurons, including those in the lateral reticular nucleus, coexpress VGluT1 and VGluT2 and project to granule cells (Li et al., 2020). In the cerebellum vermis, mossy fiber terminals show heterogeneous VGluT expression, with spinocerebellar fibers and dorsal column fibers preferentially expressing VGluT2 and VGluT1, respectively, forming complementary parasagittal bands in the granule cell layer (Gebre et al., 2012). In the mouse vestibulocerebellum, mossy fiber terminals that originated from calretinin-positive UBCs are positive for VGluT1 and VGluT2, while those originating from mGluR1α-positive UBCs are positive for VGluT1 only (Nunzi et al., 2003). This molecular diversity suggests that granule cells receive inputs with distinct physiological properties and potentially divergent functional roles. Despite these insights, it remains unclear whether granule cells integrate molecularly distinct mossy fiber types through structured or stochastic sampling.

**FIGURE 1:**
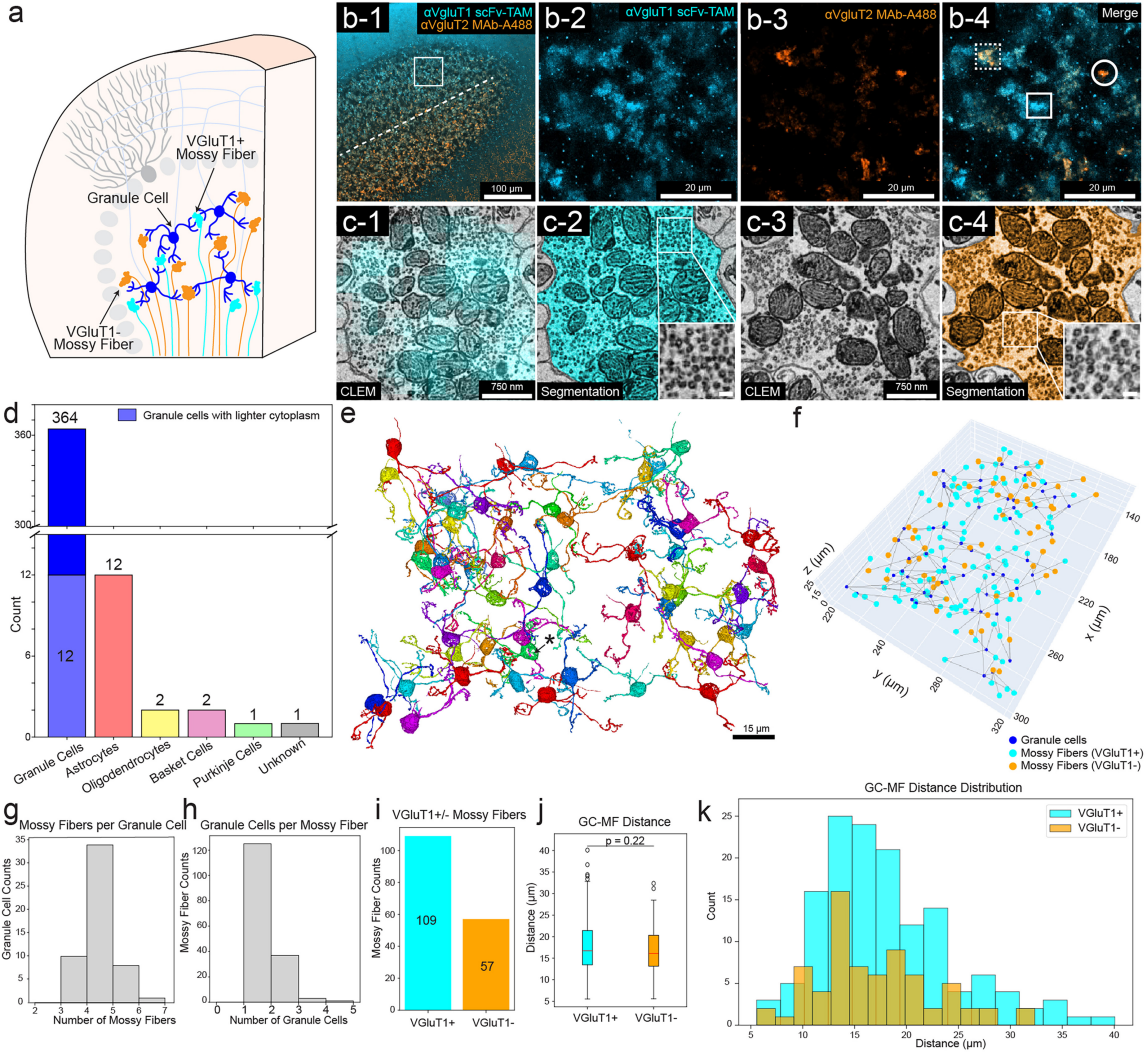
Granule cell layer composition and connectivity with VGluT1^+^ and VGluT1^–^ mossy fiber (MF) terminals. **(a)** Schematic of granule cells (GCs) and their dendritic claws (blue) connecting VGluT1^+^ (cyan) and VGluT1^–^ (orange) mossy fiber (MF) terminals in the cerebellar granule layer in the published vCLEM dataset (Han et al., 2024). **(b-1)** Immunolabeling of VGluT1 (cyan) and VGluT2 (orange) in cerebellar hemisphere Crus I, showing complementary expression across the midline (dashed line). **(b-2–b-4)** Enlarged single- and dual-channel images from the boxed region in panel **(b-1)**. Solid box: VGluT1^+^ MF terminals; dashed box: VGluT1^+^/VGluT2^+^ MF terminals; circle: VGluT2^+^ MF terminals. VGluT1^+^ boutons include both VGluT1^+^/VGluT2^+^ and VGluT1^+^/VGluT2^–^, while VGluT1^–^ boutons are VGluT2^+^ only. VGluT1^+^ terminals are much more abundant than VGluT1^–^ terminals. Reused from Han et al. (2024) with the authors’ permission. **(c-1–c-4)** CLEM images of individual MF terminals from the published dataset (Han et al., 2024). **(c-1,c-2)** VGluT1^+^ MF terminals identified by overlapping VGluT1 immunolabeling fluorescence (cyan), segmented in cyan, with inset showing synaptic vesicles. **(c-3,c-4)** VGluT1^–^ MF terminals lacking overlapping VGluT1 immunolabeling fluorescence, segmented in orange, with inset showing synaptic vesicles. **(d)** Cell type counts from the reconstructed volume: 364 granule cells (blue), 12 astrocytes, 2 oligodendrocytes, 2 basket cells, 1 Purkinje cell, and 1 unidentified cell. A subset of 12 granule cells displayed lighter cytoplasm. **(e)** 3D reconstructions of 53 granule cells fully contained in the dataset, each shown in a distinct color. Asterisks mark unlabeled regions in the automated segmentation. **(f)** Spatial map of center-of-mass coordinates of 53 granule cells (blue) and their 166 presynaptic MF terminals (VGluT1^+^: cyan; VGluT1^–^: orange). Black lines connect synaptically paired GC–MF partners. Interactive version in Supplementary materials. **(g,h)** Distributions of panel **(g)** number of MFs per GC and **(h)** number of GC per MF. **(i)** Counts of VGluT1^+^ and VGluT1^–^ boutons among the 166 reconstructed MFs. **(j)** Distances between GC–MF partners for VGluT1^+^ vs. VGluT1^–^ boutons. Welch’s *t*-test, *p* = 0.22. **(k)** Distribution of GC–MF distances for VGluT1^+^ and VGluT1^–^ boutons.

Advances in large-scale volume electron microscopy (vEM) (Peddie et al., 2022) and correlated light and electron microscopy (CLEM) (de Boer et al., 2015) now enable precise reconstruction of neuronal connectivity while simultaneously capturing molecular identity. This capability is particularly powerful for studying cerebellar granule cell inputs, where mossy fibers differ not only anatomically but also in VGluT expression. Here, we leverage a volumetric CLEM (vCLEM) dataset from the adult mouse cerebellum (Han et al., 2024) in which VGluT1-positive and VGluT1-negative mossy fiber terminals are molecularly distinguished (Figure 1a). Using this dataset, we reconstructed granule cells and their mossy fiber inputs and developed spatially constrained null models to simulate input sampling under distinct anatomical constraints.

To capture both mature connectivity and the potential developmental origins of granule cell input structure, we modeled two spatial regimes. The first reflects adult spatial constraints, based on the average dendritic reach of granule cells. The second represents a permissive upper bound on dendrites’ spatial reach opportunity during development, implemented using the maximum dendritic reach. This permissive upper-bound model is motivated by several features of granule cell development. *In vivo* imaging has shown that during early postnatal stages (P11-P23), granule cells transiently exhibit supernumerary immature dendritic processes that are later pruned as claw-like dendrites stabilize (Dhar et al., 2018). Population-level analyses indicate that overall dendritic spread is generally comparable across P10-P118, with some granule cells at earlier developmental stages exhibiting slightly broader spread (Houston et al., 2017). In parallel, mossy fibers enter the internal granule layer early, before most granule cells complete the downward migration from the external granule layer and dendritic maturation (Hámori and Somogyi, 1983; Altman, 1972; Palay and Chan-Palay, 1974; Komuro and Rakic, 1998). Together, these processes imply that granule cells may explore a larger spatial domain and encounter a broader pool of potential mossy fiber terminal partners than what is ultimately retained in the adult circuit. Accordingly, the permissive upper-bound model is not intended as a literal representation of developmental dendritic reach, but rather as a sensitivity analysis reflecting the developmental processes. We analyzed input sampling patterns using both granule cell–centered and mossy fiber–centered frameworks to assess whether either side of the synapse demonstrates non-random wiring behavior.

Our results reveal that granule cells in the adult cerebellum sample mossy fiber inputs in a significantly more structured manner than predicted by random spatial sampling. Subpopulations of granule cells preferentially sampled either VGluT1-positive or VGluT1-negative terminals. In contrast, mossy fiber terminals distributed their outputs to granule cells in a manner largely consistent with random sampling, although with subtle input-type–specific differences. These findings demonstrate that the integration of molecularly distinct mossy fiber inputs by granule cells is not random, but shaped by both spatial and developmental processes, providing new insight into the structural rules governing cerebellar microcircuit computation.

## Materials and methods

### vCLEM dataset

All analyses were performed on a previously published volumetric correlated light and electron microscopy (vCLEM) dataset from the Crus I region of the cerebellar hemisphere of an adult female mouse (Han et al., 2024). In this dataset, mossy fiber terminals were molecularly distinguished using anti-VGluT1 single-chain variable fragment (scFv) immunolabeling, enabling classification of boutons as VGluT1-positive or VGluT1-negative. The dataset was acquired using correlated confocal fluorescence imaging and serial-section scanning electron microscopy, followed by automated stitching, alignment, and 3D segmentation with manual proofreading. Full details of dataset acquisition and processing are provided in Han et al. (2024).

### Cell reconstruction and classification

A total of 382 cells were reconstructed within the middle plane of the dataset by manually assembling segments assigned to the same cell from the automated 3D segmentation. Cell types were classified according to established ultrastructural features (Palay and Chan-Palay, 1974). Of these, 364 were identified as granule cells, and the remainder included astrocytes, oligodendrocytes, basket cells, one Purkinje cell, and one cell with unknown identity. For connectivity analysis, we use the subset of 53 granule cells whose dendritic claws terminated exclusively on mossy fiber terminals fully contained within the image volume. Mossy fiber terminals were classified as VGluT1-positive or VGluT1-negative according to immunolabeling in the vCLEM dataset.

### Granule cell-mossy fiber terminal connectivity analysis

Granule cell–mossy fiber synaptic connections were identified by two ultrastructural features: (1) dendritic claws of granule cells encircling mossy fiber terminals, and (2) synaptic contacts characterized by clustered vesicles at the presynaptic terminal in electron microscopy images. Center-of-mass coordinates were manually annotated for both granule cell soma and mossy fiber terminals. Dendritic reach is defined as the Euclidean distance between the center of mass of each granule cell soma and the center of mass of its connected mossy fiber terminal(s). Summary statistics include mean, standard deviation (SD), and maximum distances.

### Null models

Two spatially constrained null models were implemented. In the adult model, a sphere with a radius equal to the average granule cell dendritic reach (17.62 μm) was centered on each cell, and connections were randomly assigned to mossy fibers whose centers of mass fell within the sphere. The number of connections was fixed to match empirical data. In a refined version of the adult model, each granule cell was assigned a cell-specific sampling radius equal to its own average dendritic reach (Supplementary Table 1). In the permissive upper-bound model to reflect developmental processes, the sphere radius was set to the maximum observed dendritic reach (40.08 μm). In a refined version of the permissive upper-bound model, each granule cell was assigned to a cell-specific sampling radius equal to its own maximum dendritic reach (Supplementary Table 2). For mossy fiber–centered analyses, spheres of equivalent radii were centered on mossy fiber terminals. Each model was run with 1,000 Monte Carlo simulations.

To assess the potential influence of boundary effects, we excluded granule cells whose soma centers lay within 17.62 μm (the mean dendritic reach) of the upper boundary of the reconstructed volume, and repeated the simulations of the adult model and the permissive upper-bound null models.

### Radius sensitivity and spatial overlap analysis

To access how spatial sampling radius influences the expected connectivity, we performed a radius sensitivity analysis in which the spatial sampling radius was varied across six values spanning the empirical range of dendritic reach (minimum, intermediate values between the minimum and average, the average reach, and intermediate values between the average and maximum, up to the maximum). For each radius, we quantified the expected fraction of granule cell-granule cell (GC-GC) pairs sharing mossy fiber terminals under the null model.

To explicitly characterize the geometric properties of the sampling model, we further quantified the overlap between granule cell sampling spheres. For each GC pair, a binary overlap metric was calculated: overlap = 1 if the distance between GC centers of mass was less than twice the sampling radius, and overlap = 0 otherwise. The fraction of GC pairs with overlapping sample spheres was then computed for each radius. In addition, we quantified the sets of mossy fiber terminal pools available to GC pairs. For each GC pair, we determined the sets of mossy fiber terminals whose centers of mass fell within the radius-defined sampling sphere of each GC, and calculated the fraction of mossy fiber terminals shared between the two sets using the Jaccard index. This overlap was computed both across all GC pairs and among GC pairs whose spatial sampling spheres overlapped.

### Statistical analysis

Distances between VGluT1-positive and VGluT1-negative granule cell-mossy fiber terminal (GC–MF) pairs were compared using Welch’s *t*-test. For comparisons between empirical measurements and null-model simulations, one-sided empirical *p*-values were computed from 1,000 Monte Carlo iterations. Specifically:

- When the empirical statistic was lower than the null mean (e.g., fraction of GC–GC pairs sharing ≥ 1 MF):
$$p=P_{\text{null}}\left(T\leq T_{\text{emp}}\right)=\frac{1}{N}\sum_{i}1\left[T_{i}\leq T_{\text{emp}}\right]$$
  implemented in code as np.mean(null_stats ≤ emp_stat).
- When the empirical statistic was higher than the null mean (e.g., fraction of GC subtype targets where real > null):
$$p=P_{\text{null}}\left(T\geq T_{\text{emp}}\right)=\frac{1}{N}\sum_{i}1\left[T_{i}\geq T_{\text{emp}}\right]$$
  implemented in code as np.mean(null_stats ≥ emp_stat).

Effect sizes were quantified as Cohen’s d relative to the null distribution, calculated as:


d=(T⁢e⁢m⁢p-μ⁢n⁢u⁢l⁢l)/σ⁢n⁢u⁢l⁢l

Differences in input fractions were assessed using Mann–Whitney U tests. Equivalent null-model comparisons and effect size calculations were performed for additional connectivity features, including the fractions of VGluT1-positive or negative terminals contacted by each GC, MF-MF pair sharing, GC subtype preference fractions, and MF target diversity. All analyses and visualizations were performed in Python. The complete Jupyter Notebook used for these analyses is provided in the Supplementary materials.

## Results

### Molecular and cellular profiling of the granule cell layer

**FIGURE 2:**
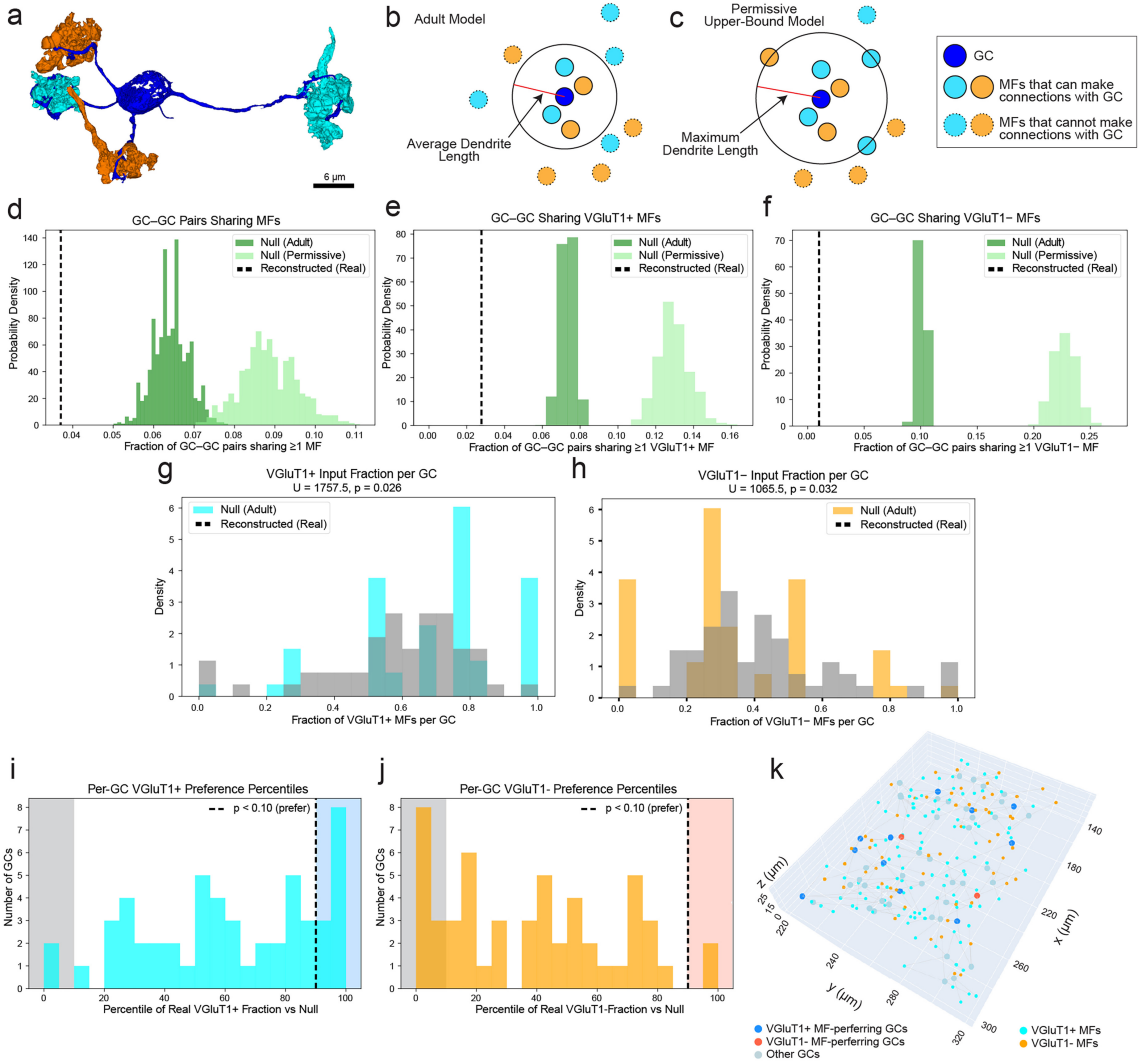
Granule cells exhibit structured, non-random sampling of mossy fiber inputs. **(a)** 3D reconstruction of a centered granule cell (blue) connected to two VGluT1^+^ (cyan) and two VGluT1^–^ (orange) mossy fiber terminals. **(b,c)** Null models of GC-centered connectivity. **(b)** Adult null model: connections assigned within a sphere defined by the average dendrite length (17.62 μm). **(c)** Permissive upper-bound null model: connections assigned within a sphere defined by the maximum dendrite length (40.08 μm). **(d–f)** Probability density distributions of the fraction of GC–GC pairs sharing ≥ 1 MF. Real data (black dashed line) are compared with null distributions from the adult (dark green) and permissive upper-bound (light green) models. **(d)** All MFs, **(e)** only VGluT1^+^ MFs, **(f)** only VGluT1^–^ MFs. **(g,h)** Fractions of VGluT1^+^ **(g)** or VGluT1^–^ **(h)** MF inputs per GC in the real dataset (cyan/orange) compared to the adult null model (gray). Mann–Whitney U test, VGluT1^+^: *U* = 1757.5, *p* = 0.026; VGluT1^–^: *U* = 1065.5, *p* = 0.032. **(i,j)** Percentile ranks of each GC’s VGluT1^+^ **(i)** or VGluT1^–^ **(j)** input fraction relative to the adult null model. **(i)** Granule cells above the 90th percentile (blue shading) were classified as VGluT1^+^-preferring. Granule cells below the 10th percentile (gray shading) correspond to the VGluT1^–^-preferring group shown in panel **(j)**. **(j)** Granule cells above the 90th percentile (blue shading) were classified as VGluT1^–^-preferring, while those below the 10th percentile (gray shading) correspond to the VGluT1^+^-preferring group in panel **(i)**. **(k)** Spatial map of the three types of GCs [VGluT1^+^ preferring (blue), VGluT1^–^ preferring (red), and the rest (gray)] and the MF terminals (VGluT1^+^: cyan; VGluT1^–^: orange). Black lines connect synaptically paired GC–MF partners. Interactive version in Supplementary materials.

**FIGURE 3:**
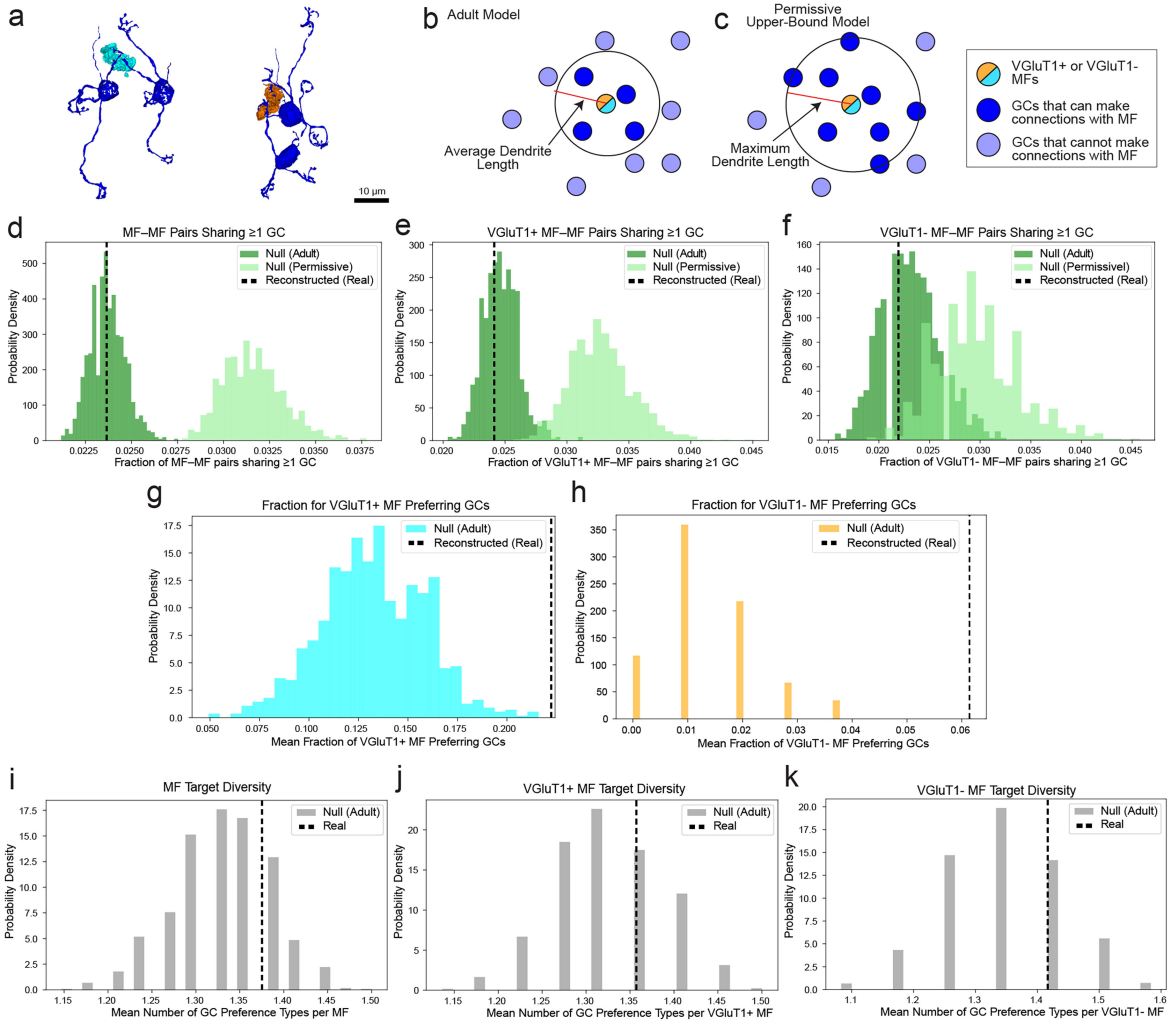
Mossy fibers distribute outputs in a largely random manner across granule cells. **(a)** 3D reconstructions of example mossy fiber terminals: a VGluT1^+^ terminal (cyan) contacting two granule cells (GCs, blue) and a VGluT1^–^ terminal (orange) contacting two GCs. **(b,c)** Null models for MF-centered connectivity. **(b)** Adult null model: connections assigned within a sphere defined by the average dendrite length (17.62 μm). **(c)** Permissive upper-bound null model: connections assigned within a sphere defined by the maximum dendrite length (40.08 μm). **(d–f)** Probability density distributions of the fraction of MF–MF pairs sharing ≥ 1 GC. Real data (black dashed line) are compared with null distributions from the adult (dark green) and permissive upper-bound (light green) models. **(d)** All MFs, **(e)** only VGluT1^+^ MFs, **(f)** only VGluT1^–^ MFs. **(g,h)** Probability density distributions of the mean fractions of VGluT1-positive-preferring **(g)** or VGluT1-negative-preferring **(h)** GC subtypes contacted by each MF in the real dataset (black dashed line) compared to the adult null model. **(i–k)** Probability density distributions of the mean target diversity, defined as the number of GC preference types (VGluT1-positive-preferring, VGluT1-negative-preferring, or non-preferring) contacted by each MF in the real dataset (black dashed line) compared to the adult null model. **(i)** All MFs, **(j)** only VGluT1^+^ MFs, **(k)** only VGluT1^–^ MFs.

We began by characterizing the molecular features of mossy fiber terminals and cellular composition of the granule cell layer within the published vCLEM dataset from an adult female mouse cerebellum (Crus 1 of the hemisphere) (Han et al., 2024). Prior analysis of this dataset revealed that VGluT1-positive mossy fiber terminals are significantly more abundant and exhibit higher synaptic vesicle density and volume than VGluT1-negative terminals (Han et al., 2024; Figures 1b-1–b-4, c-1–c-4).

We reconstructed a total of 382 cells located within the middle plane of the dataset (Supplementary Figure 1a). Among these, 364 were identified as granule cells, while the remainder included 12 astrocytes, 2 oligodendrocytes, 2 basket cells, 1 Purkinje cell, and 1 cell of unknown identity (Figure 1d).

Among the 364 granule cells, a subset of 12 granule cells displayed lighter cytoplasmic contrast in EM images (Figure 1d, Supplementary Figure 1b), though the functional significance of this feature remains unclear. Among the 364 granule cells, we identified 53 in which all dendritic claws terminated on mossy fiber boutons fully contained within the image volume (Figure 1e). These 53 cells formed synapses with 166 mossy fiber terminals, including 109 VGluT1-positive and 57 VGluT1-negative boutons (Figures 1f, i). Each granule cell contacted an average of 4.00 ± 0.65 (mean ± SD) mossy fiber terminals (Figure 1g). Conversely, each mossy fiber terminal contacted an average of 1.28 ± 0.52 (mean ± SD) granule cells (Figure 1h). We measured the dendritic reach, defined as the Euclidean distance between the centers of mass of each granule cell and its connected mossy fiber terminals. The overall average dendritic reach was 17.62 ± 6.22 (mean ± SD) μm, with a maximum of 40.08 μm. For VGluT1-positive terminals, the mean dendritic reach was 17.98 ± 6.44 (mean ± SD) μm (maximum: 40.08 μm) (Figure 1j), while VGluT1-negative terminals showed a slightly shorter average dendritic reach of 16.91 ± 5.74 (mean ± SD) μm (maximum: 32.37 μm) (Figure 1j). These values are consistent with prior measurements of granule cell dendritic reach (Nguyen et al., 2022), suggesting that while our sample may favor cells with shorter dendrites due to volume constraints, it remains representative. No significant differences were observed in the distances of VGluT1-positive versus VGluT1-negative terminals (Welch’s *t*-test, *p* = 0.22; Figures 1j, k). The center-of-mass coordinates of this set of 53 fully reconstructed granule cells and their 166 mossy fiber partners (Figure 1f) formed the basis for the quantitative modeling and connectivity analyses described below.

### Granule cell-centered analysis reveals structured sampling of mossy fiber inputs

To assess whether granule cells sample mossy fiber inputs randomly or in a structured manner, we built two spatially constrained null models centered on each granule cell (Figure 2a). The first model (Figure 2b; Adult null model) simulated the adult state by constructing a sphere centered at each granule cell using the average dendritic reach (17.62 μm) of the 53 complete cells as its radius, and randomly assigned connections to mossy fiber terminals whose centers of mass fell within the sphere. The number of connections was fixed to match the empirical data (Figure 1g applied). In a refined version of the adult model, a cell-specific average dendritic reach was used (see Supplementary Table 1). The second model (Figure 2c; Permissive upper-bound null model) represents a sensitivity analysis that reflects developmental processes by using the maximum dendritic reach (40.08 μm) observed among the 53 granule cells as the sphere’s radius. In a refined version of the permissive upper-bound model, a cell-specific maximum dendritic reach was used (see Supplementary Table 2). For both models, we ran 1,000 Monte Carlo simulations.

We first compared the number of granule cell pairs that share at least one mossy fiber input between the empirical data and the null models. The number of shared mossy fiber partners in the real dataset was significantly lower than in both the adult and permissive upper-bound null models [Figure 2d; Adult null model, *p*-value (real < null) = 0.0000, Cohen’s *d* = −6.254; Permissive upper-bound null model, *p*-value (real < null) = 0.0000, Cohen’s *d* = −7.408]. This suggests that granule cells sample mossy fiber terminals in a structured manner rather than through purely random selection. Furthermore, the deviation (Figure 2d, measured by Cohen’s d values) was greater in the adult model than in the permissive upper-bound model, indicating that selective synapse making or pruning from development to adulthood may enhance this structured sampling. The refined version of the adult model and the permissive upper-bound model showed qualitatively similar results (Supplementary Figures 2a, 3a). Repeating the analyses of the adult and permissive upper-bound models after excluding granule cells near the upper boundary of the reconstructed volume did not qualitatively alter the results (Supplementary Figure 4a).

To examine whether these results depend on the specific choice of sampling radius, we performed a radius-sensitivity analysis in which the null model was evaluated across six radii spanning the empirical range of dendritic reach. As expected from geometric considerations, the null prediction showed a monotonic increase in the expected sharing of mossy fiber terminals with the increasing radius (Supplementary Figure 5a). In contrast, the empirical value remained constant and diverged from the null expectation across most biologically relevant radii, indicating that the observed connectivity cannot be explained solely by distance-limited random sampling.

To explicitly characterize the geometric baseline of the spatial sampling model, we quantified the overlap between granule cell sampling spheres across the same range of radii. As expected, the fraction of the overlapping granule cell pairs increased with sampling radius (Supplementary Figure 5b), as did the overlap between candidate mossy fiber terminal pools available to granule cell pairs (Supplementary Figure 5c). These analyses confirm that spatial geometry strongly influences the expected probability of shared mossy fiber terminals under the null model.

When broken down the mossy fibers into two categories (VGluT1-positive and VGluT1-negative), we observed the number of shared mossy fiber partners in the real dataset was significantly lower than in both the adult and permissive upper-bound models [Figure 2e, VGluT1-positive MFs; Adult null model, *p*-value (real < null) = 0.0000, Cohen’s *d* = −12.711; Permissive upper-bound null model, *p*-value (real < null) = 0.0000, Cohen’s *d* = −12.140] [Figure 2d, VGluT1-negative MFs; Adult null model, *p*-value (real < null) = 0.0000, Cohen’s *d* = −26.482; Permissive upper-bound null model, *p*-value (real < null) = 0.0000, Cohen’s *d* = −21.476] no matter if the mossy fiber terminal is VGluT1-positive or VGluT1-negative. We also observed a stronger deviation from the null models for VGluT1-negative terminals, as quantified by Cohen’s d (Figures 2e, f). This supports the hypothesis that granule cells may adopt a strategy to more effectively sample sparser input types such as VGluT1-negative mossy fibers. Again, the refined version of the adult and permissive upper-bound models showed qualitatively similar results (Supplementary Figures 2b, c, 3b, c). Results were qualitatively unchanged after excluding granule cells near the upper boundary (Supplementary Figures 4b, c).

We next quantified the fraction of VGluT1-positive and VGluT1-negative terminals contacted by each granule cell. Compared to the adult null model, the real dataset showed an overrepresentation of GCs strongly biased toward VGluT1-positive inputs, as well as a smaller group biased toward VGluT1-negative inputs (For VGluT1-positive MF fraction, Mann–Whitney U test, *U* = 1757.5, *p* = 0.026 < 0.05) (For VGluT1-negative MF fraction, Mann–Whitney U test, *U* = 1065.5, *p* = 0.032 < 0.05) (Figures 2g, h). Note that the results in Figures 2g, h were generated from two independent Monte Carlo simulations and therefore showed small stochastic differences. For comparison, results from a single Monte Carlo simulation, in which that VGluT1-positive and VGluT1-negative fractions are exactly opposite each other, were shown in Supplementary Figures 6a, b.

Using the adult null model we quantified the percentile rank of each granule cell’s fraction of VGluT1-positive MF inputs relative to random sampling. Granule cells whose real fraction exceeded the 90th percentile of their null distribution were classified as VGluT1-positive-preferring (Figure 2i). Conversely, applying the same procedure to VGluT1-negative MFs identified VGluT1-negative-preferring granule cells (Figure 2j). Notably, cells that ranked in the top decile for VGluT1-positive input preference consistently fell in the bottom decile for VGluT1-negative preference, and vice versa (Figures 2i, j), reinforcing the existence of structured, non-random sampling subtypes. Based on these classifications, we divided the population into three groups: VGluT1-positive-preferring (*n* = 11), VGluT1-negative-preferring (*n* = 2), and non-preferring/neutral (*n* = 40) granule cells (see Figure 2k for their spatial distribution). Again, the results in Figures 2i, j were generated from two independent Monte Carlo simulations. Results from a single Monte Carlo simulation, in which that VGluT1-positive and VGluT1-negative distributions are exactly opposite each other, were shown in Supplementary Figure 6c.

### Mossy fiber-centered analysis suggests random distribution of output

We next examined mossy fiber terminal output patterns using a complementary null model approach centered on each mossy fiber terminal (Figure 3a). The first model (Figure 3b) simulated the adult state by constructing a sphere centered at each mossy fiber terminal using the average dendritic reach (17.62 μm), and randomly assigned connections to granule cells whose centers of mass fell within the sphere. The number of connections was fixed to match the empirical data (Figure 1h applied). In a refined version of the adult model, a granule cell-specific average dendritic reach was used (see Supplementary Table 1). The second permissive upper bound model (Figure 3c), as stated before, represents an analysis that reflects developmental processes by using the maximum dendritic reach (40.08 μm). Again, in a refined version of the permissive upper-bound model, a granule cell-specific maximum dendritic reach was used (see Supplementary Table 2). For both models, we ran 1,000 Monte Carlo simulations.

We compared the fraction of mossy fiber pairs that shared at least one granule cell target in the empirical data to those in the null models. The observed overlap was not significantly different from the adult null model [Figure 3d; Adult null model, *p*-value (real < null) = 0.5340, Cohen’s *d* = −0.032] but was significantly lower than the Permissive upper-bound model [Figure 3d; Permissive upper-bound null model, *p*-value (real < null) = 0.0000, Cohen’s *d* = −4.898]. Interestingly, when spatial heterogeneity in granule cell dendritic reach was incorporated by assigning each granule cell its empirically measured average dendritic reach (in the refined adult model), the empirical sharing became significantly lower than expected [Supplementary Figure 2d; Refined adult null model, *p*-value (real < null) = 0.0090, Cohen’s *d* = −2.258]. Refined permissive upper-bound model showed quantitatively similar results to the permissive upper-bound model [Supplementary Figure 3d; Refined permissive upper-bound null model, *p*-value (real < null) = 0.0000, Cohen’s *d* = −4.921]. Excluding granule cells near the upper boundary did not qualitatively change the results [Supplementary Figure 4d; Permissive upper-bound null model, *p*-value (real < null) = 0.0000, Cohen’s *d* = −4.263]. This indicates that mossy fibers distribute their outputs in a largely random manner in adulthood, with selective synapse making or pruning from development to adulthood likely contributing to the emergence of weak selectivity.

Because individual mossy fiber terminals in our dataset contact only a small number of granule cells on average (mean = 1.28), the MF-centered analysis may have reduced statistical sensitivity to detect deviations from random sampling. To address this potential limitation, we repeated the MF-centered analysis using only mossy fiber terminals that contacted two or more granule cells in the reconstructed dataset. Restricting the analysis to this more informative subset did not reveal significant deviation from the null expectation [Supplementary Figure 7a; Adult null model, *p*-value (real < null) = 0.3040, Cohen’s *d* = −0.702; Permissive upper-bound null model, *p*-value (real < null) = 0.0000, Cohen’s *d* = −2.502].

Breaking the analysis down by molecular identity, we found that VGluT1-positive terminals behaved similarly to the overall population [Figure 3e, VGluT1-positive MFs; Adult null model, *p*-value (real < null) = 0.3960, Cohen’s *d* = −0.374; Permissive upper bound null model, *p*-value (real < null) = 0.0000, Cohen’s *d* = −3.413]. However, in the refined adult model, VGluT1-positive terminals again showed a significant deviation from the null expectation [Supplementary Figure 2e; Refined adult null model, *p*-value (real < null) = 0.0320, Cohen’s *d* = −1.793], consistent with the trend observed in the full mossy fiber terminal population. VGluT1-negative terminals behaved similarly to the overall population in adult or permissive upper bound null model [Figure 3f, VGluT1-negative MFs; Adult null model, *p*-value (real < null) = 0.4130, Cohen’s *d* = −0.398; Permissive upper bound null model, *p*-value (real < null) = 0.0150, Cohen’s *d* = −1.897], but the refined adult model produced a non-significant trend from the null expectation [Supplementary Figure 2f; Refined adult null model, *p*-value (real < null) = 0.0770, Cohen’s *d* = −1.426]. Again, the refined permissive upper-bound models showed qualitatively similar results for both VGluT1-positive and -negative terminals to the permissive upper-bound model (Supplementary Figures 3e, f), including after excluding granule cells near the upper boundary (Supplementary Figures 4e, f). These observations suggest that VGluT1-positive terminals largely follow the same spatial organization observed in the full mossy fiber terminal population, whereas VGluT1-negative terminals exhibit a slightly weaker deviation from random sampling. This suggests that VGluT1-negative terminals exhibit a more random distribution than VGluT1-positive terminals, potentially as a strategy to ensure granule cells’ broad access to these sparser inputs.

We further evaluated whether mossy fibers preferentially target specific granule cell subtypes as defined in the previous section. We compared the fraction of VGluT1-positive-preferring (Figure 3g) or VGluT1-negative-preferring (Figure 3h) granule cells contacted by each mossy fiber terminal to the adult null model. The observed values were significantly higher than expected by chance [Figures 3g, h; For VGluT1-positive-preferring GC fraction, *p*-value (real > null) = 0.0000; For VGluT1-negative-preferring GC fraction, *p*-value (real > null) = 0.0000], indicating that mossy fibers do bias connectivity toward granule cells predisposed to sample them.

Finally, we assessed the target diversity of granule cell targets across terminals by classifying GCs into three types: VGluT1-positive-preferring, VGluT1-negative-preferring, or others with the criterion of the top and bottom 10%. The target diversity value is defined by how many subtypes of GC contacted by a MF (1, 2, or 3). For all terminals, including VGluT1-positive and VGluT1-negative subsets, we found that the diversity of GC target types did not significantly differ from the adult null model [Figures 3i–k; All MF target diversity, *p*-value (real > null) = 0.238; VGluT1-positive MF target diversity, *p*-value (real > null) = 0.399; VGluT1-negative MF target diversity, *p*-value (real > null) = 0.341]. This supports the idea that, while some degree of preferential targeting exists, mossy fiber terminals generally adopt a strategy to maximize distribution across available granule cells.

## Discussion

Our findings provide new insight into the structural logic of granule cell connectivity within the cerebellar cortex, demonstrating that granule cells sample mossy fiber inputs in a non-random, structured manner. Using molecularly defined terminals and spatially constrained null models, we revealed that granule cells exhibit input preferences that are stronger than expected by chance, particularly with respect to VGluT1-negative terminals, which are less abundant in this region (Han et al., 2024). This suggests that granule cells may compensate for input sparsity by adopting a broader sampling strategy for less common input types.

The contrast between granule-cell–centered and mossy-fiber–centered analyses highlights an important asymmetry in circuit organization. While granule cells appear selective in their sampling, mossy fiber terminals distribute their outputs in a manner largely consistent with random targeting. This discrepancy implies that input selection may be driven more by granule cell properties, such as dendritic reach or developmental pruning, rather than by targeted output from mossy fibers (Altman and Bayer, 1978; Komuro and Rakic, 1995, 1998; Kim M. et al., 2023; Schuldiner and Yaron, 2015; Lackey et al., 2018). This also corresponds to the fact that during development, mossy fibers enter the granule layer and form terminals before granule cells descend into the granule layer (Schilling et al., 1994; Hámori and Somogyi, 1983; Kim T. et al., 2023; Altman, 1972). These observations suggest that granule cell properties may play a dominant role in shaping synaptic partner selection. However, this apparent asymmetry should be interpreted cautiously because mossy fiber terminals in our dataset contact relatively few granule cells on average. This limited connectivity divergence reduced the statistical power of mossy fiber terminal-centered analyses to detect deviations from random connectivity and, therefore, may mask potential structure in mossy fiber output patterns.

Our modeling across adult and permissive upper-bound spatial regimes indicated that the observed granule cell-mossy fiber connectivity cannot be explained by spatial accessibility alone. The empirical data deviated from both models, demonstrating robust non-random input sampling, consistent with a pruning or competitive synapse-making mechanism that enhances input diversification among neighboring granule cells (Hámori and Somogyi, 1983; Lackey et al., 2018; Kim M. et al., 2023; Dhar et al., 2018). Differences in deviation magnitudes between the adult and permissive models are expected to depend on geometric overlap and are therefore not interpreted mechanistically.

An interesting insight emerged when spatial heterogeneity in granule cell dendritic reach was incorporated into the null model for mossy-fiber-centered analysis. When all granule cells were allowed to sample within a radius equal to the global average dendritic reach (the adult null model), the observed frequency of mossy fiber terminal sharing more than one granule cell was indistinguishable from random spatial sampling. However, when each granule cell was assigned to its empirical average dendritic reach (the refined adult null model), the observed degree of granule cell sharing by mossy fiber terminal pairs became significantly lower than random sampling. This difference arises because variability in dendritic reach increases overlap among candidate granule cell pools accessible to different mossy fiber terminals, thereby increasing the probability that the same mossy fiber terminal pair would converge onto multiple granule cells under random sampling. The fact that the empirical connectivity falls below this prediction suggests that mossy fiber terminals do not fully sample all spatially accessible granule cells. Instead, the circuit appears to modestly limit redundant convergence onto the same granule cell pairs, a similar pattern observed in our granule-cell-centered analysis. Consistent with this interpretation, the same was observed from VGluT1-positive mossy fiber terminals, whereas VGluT1-negative terminals showed a similar but weaker deviation from the null expectation. The slightly more random sampling of granule cells by VGluT1-negative terminals may reflect a strategy to ensure adequate integration of underrepresented input streams (Gebre et al., 2012; Nunzi et al., 2003).

Several limitations should be considered when interpreting our findings. First, the distance-limited null models we used here provide a simplified description of synaptic partner selection. Although these models preserve the empirical spatial arrangement of granule cells and mossy fiber terminals, they test only whether connectivity can be explained by spatial accessibility under random sampling. Other biological factors, including molecular, developmental, and activity-dependent factors, are not modeled and may contribute to the observed connectivity. Second, the reconstructed volume represents only a small portion of the cerebellar cortex. Consequently, many fiber terminals and granule cell dendrites likely extend beyond the dataset’s boundaries, limiting the number of mossy fiber terminals’ partners captured and potentially reducing the statistical power of mossy fiber terminal-centered analyses. In addition, the finite boundaries of our reconstructed volume may introduce geometric biases. For example, granule cells located near volume borders, particularly adjacent to the Purkinje cell layer, may exhibit non-uniform dendrite distribution, with dendrites preferentially oriented downward to the interior of the granule layer. Such directional bias could restrict the local pool of possible mossy fiber terminal partners. However, our additional analysis excluding granule cells near the upper boundary (next to the Purkinje cell layer) of the volume yielded qualitatively consistent results, indicating that at least the non-uniform distribution of dendrites adjacent to the Purkinje cell layer does not account for the observed non-random sampling. Future studies using larger-scale EM volumes with more reconstructed granule cells, together with finer analysis on dendritic spatial uniformity, will be well positioned to examine how dendritic growth patterns contribute to the non-random mossy fiber input sampling strategy by granule cells.

Recent connectomics studies using vEM also demonstrate that granule cell input sampling is not entirely random. For example, previous work reports that granule cells share mossy fiber partners more frequently than predicted by spatially constrained random models, and individual mossy fibers are unevenly represented across the granule cell population (Nguyen et al., 2022). In contrast, our analysis revealed the opposite trend: granule cells in our vCLEM dataset shared mossy fiber partners less often than expected under equivalent spatially constrained null models. This discrepancy may reflect differences in the cerebellar regions examined [vermis in Nguyen et al. (2022) vs. Crus I in our study]. Moreover, the underrepresented and overrepresented mossy fiber terminals described by Nguyen et al. (2022) may correspond to distinct molecular subtypes, a distinction that is not accessible in their dataset because molecular identity was not labeled. By incorporating molecular identity through vCLEM, we extend these findings and demonstrate that structured sampling is especially evident for VGluT1-negative inputs. These results align with theoretical predictions that structured synaptic connectivity in the granule cell layer enhances pattern separation and expansion coding (Litwin-Kumar et al., 2017; Cayco-Gajic et al., 2017).

Because our analyses identified deviations from spatially random connectivity but did not fully resolve the circuit mechanisms underlying the structure, future studies will be needed to determine how these sampling rules arise. The presence of granule cell subtypes with distinct input preferences suggests a potential basis for functional heterogeneity within the granule cell layer (Masoli et al., 2020; De Zeeuw et al., 2021). These subtypes may contribute to parallel processing streams (Ishikawa et al., 2015) or encode different aspects of sensorimotor information (Knogler et al., 2017; Sylvester et al., 2017), a hypothesis that warrants future investigation using other experimental approaches such as functional imaging or electrophysiology.

Our analysis was based on the published vCLEM dataset from the cerebellar Crus1 hemisphere, owing to the possibility of overlaying molecular identity on EM data with CLEM. To expand this type of analysis and deepen our understanding of how granule cells sample distinct mossy fiber inputs, generating more such correlative datasets on larger scales would be beneficial. Dye-based (Holtzman et al., 2009) or virus-based fluorescence tracing techniques (Pisano et al., 2021; Dohaku et al., 2019) can be combined to label mossy fibers from distinct regions. Furthermore, newer light microscopy-based connectomics approaches such like LICONN (Tavakoli et al., 2025) can help with accumulating more connectivity data with molecular identification.

Altogether, this study establishes a framework for examining how cerebellar microcircuits are wired to balance input molecular diversity, spatial constraints, and developmental process. Our findings provide a framework for examining how spatial constraints, molecular identity, and developmental processes jointly shape granule cell input sampling and cerebellar computation.

## Data Availability

The datasets presented in this study can be found in online repositories. The names of the repository/repositories and accession number(s) can be found in the article/Supplementary material, and Han et al. (2024).
