# Supplementary material for: Structured sampling of molecularly classified mossy fiber inputs by cerebellar granule cells
Source: Front Comput Neurosci. 2026 Jun 22;20:1717379. doi: 10.3389/fncom.2026.1717379 (PMC13333720; doi:10.3389/fncom.2026.1717379)
Supplement: Supplementary file 2 [file Data_Sheet_1.docx]

**Structured Sampling of Molecularly Classified Mossy Fiber Inputs by Cerebellar Granule Cells**

**Supplementary Materials**

Xiaomeng Han^1*^, Elif Sevde Meral^2^, Jeff Lichtman^1*^

1 Department of Molecular and Cellular Biology, Harvard University, Cambridge, MA

2 Bezmialem Vakif University School of Medicine, Istanbul, Turkey

* Corresponding authors

**
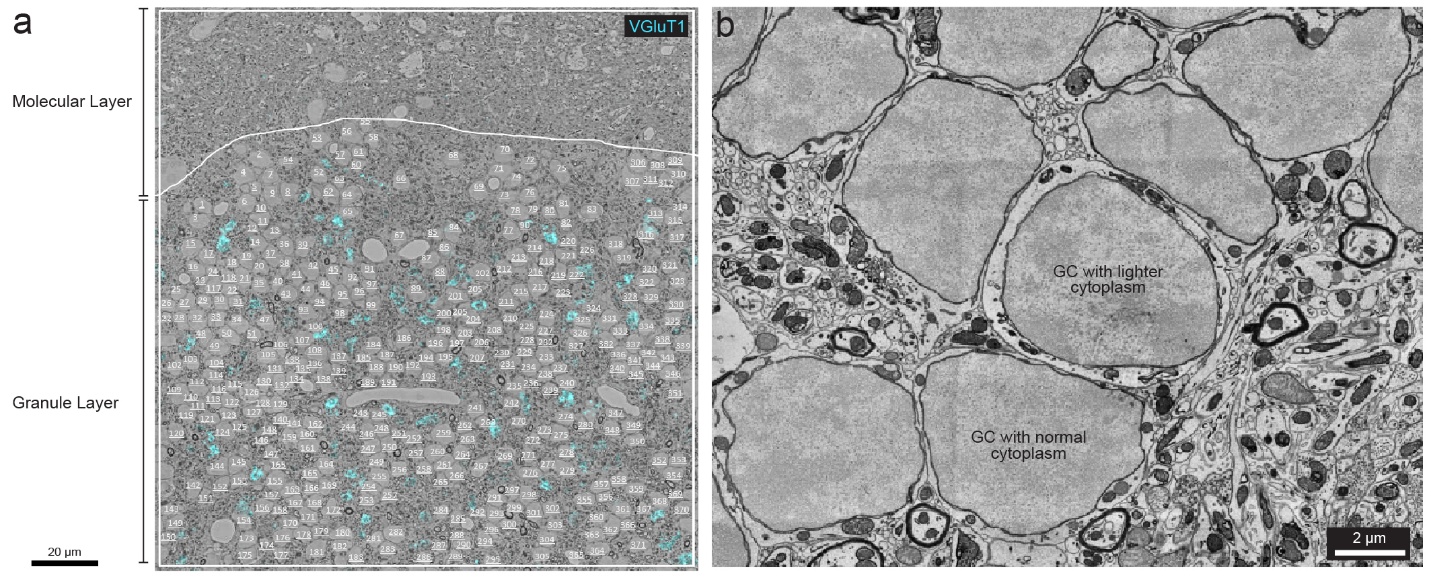
**

**Sup. Figure 1. Additional features of the granule cell layer in the vCLEM dataset.**

(**a**) Locations of 382 reconstructed cells (white labels) within the middle plane of the volume.

(**b**) EM micrograph showing examples of granule cells with normal versus lighter cytoplasm.

**
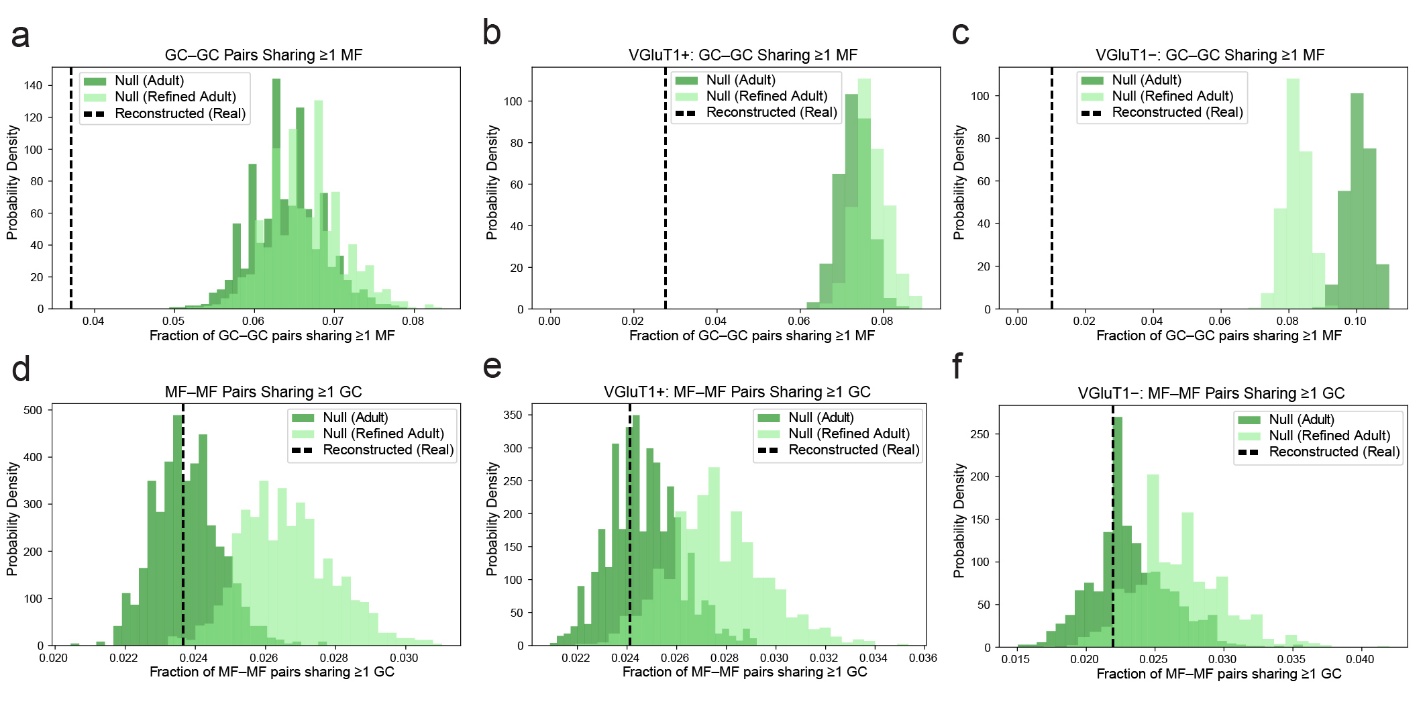
**

**Sup. Figure 2. Results from the refined adult models.**

(a–c) Probability density distributions of the fraction of GC–GC pairs sharing ≥1 MF. Real data (black dashed line) are compared with null distributions from the adult (dark green) and refined adult (light green) models. (a) All MFs. Adult null model, p-value (real < null) = 0.0000, Cohen's d = -6.137; Refined adult null model, p-value (real < null) = 0.0000, Cohen's d = -6.350. (b) only VGluT1+ MFs. Adult null model, p-value (real < null) = 0.0000, Cohen's d = -12.459; Refined adult null model, p-value (real < null) = 0.0000, Cohen's d = -12.288. (c) only VGluT1– MFs. Adult null model, p-value (real < null) = 0.0000, Cohen's d = -25.767; Refined adult null model, p-value (real < null) = 0.0000, Cohen's d = -20.923.

(d-f) Probability density distributions of the fraction of MF–MF pairs sharing ≥1 GC. Real data (black dashed line) are compared with null distributions from the adult (dark green) and refined adult (light green) models. (d) All MFs. Adult null model, p-value (real < null) = 0.4980, Cohen's d = -0.090; Refined adult null model, p-value (real < null) = 0.0090, Cohen's d = -2.258. (e) only VGluT1+ MFs. Adult null model, p-value (real < null) = 0.3910, Cohen's d = -0.402; Refined adult null model, p-value (real < null) = 0.0320, Cohen's d = -1.793. (f) only VGluT1– MFs. Adult null model, p-value (real < null) = 0.3940, Cohen's d = -0.427; Refined adult null model, p-value (real < null) = 0.0770, Cohen's d = -1.426.

**
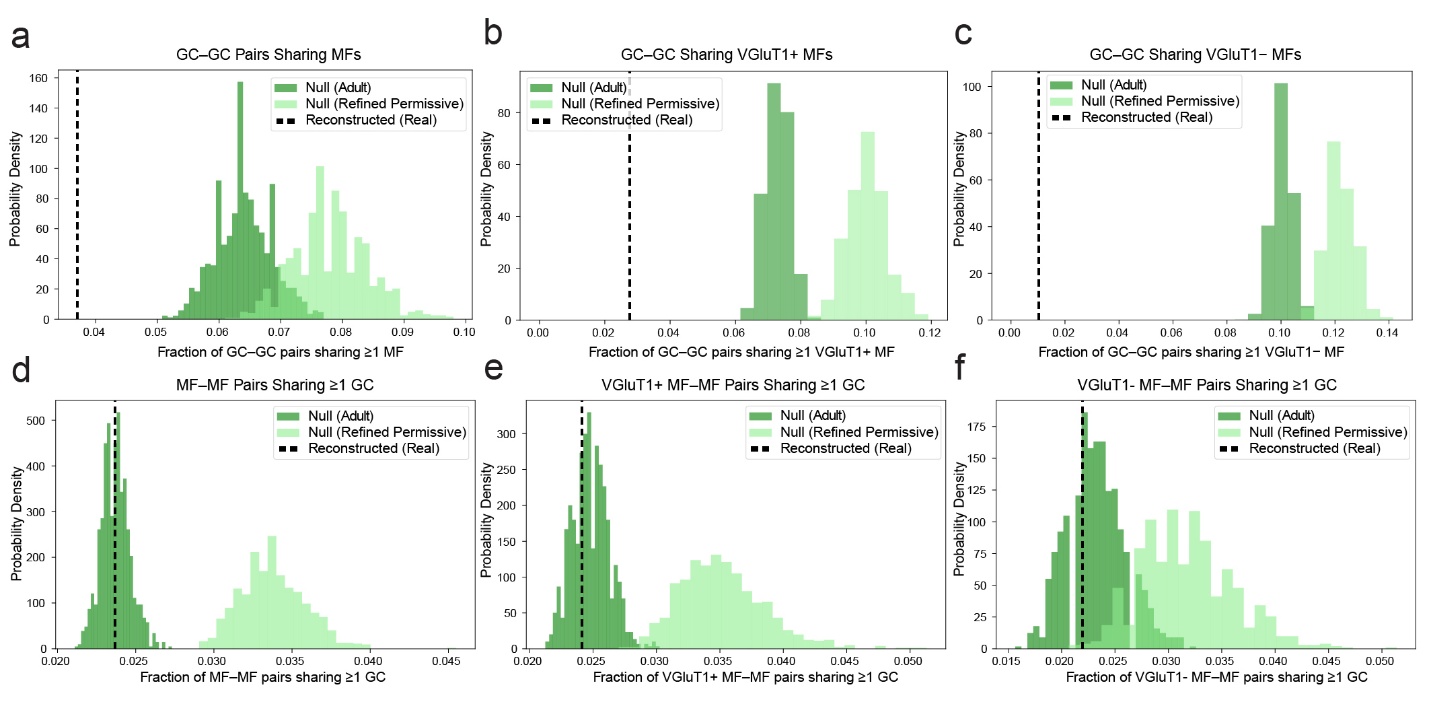
**

**Sup. Figure 3. Results from the refined permissive upper-bound models.**

(a–c) Probability density distributions of the fraction of GC–GC pairs sharing ≥1 MF. Real data (black dashed line) are compared with null distributions from the adult (dark green) and refined permissive upper-bound (light green) models. (a) All MFs. Adult null model, p-value (real < null) = 0.0000, Cohen's d = -6.020; Refined permissive upper-bound null model, p-value (real < null) = 0.0000, Cohen's d = -6.961. (b) only VGluT1+ MFs. Adult null model, p-value (real < null) = 0.0000, Cohen's d = -12.711; Refined permissive upper-bound null model, p-value (real < null) = 0.0000, Cohen's d = -12.903. (c) only VGluT1– MFs. Adult null model, p-value (real < null) = 0.0000, Cohen's d = -26.482; Refined permissive upper-bound null model, p-value (real < null) = 0.0000, Cohen's d = -12.240.

(d-f) Probability density distributions of the fraction of MF–MF pairs sharing ≥1 GC. Real data (black dashed line) are compared with null distributions from the adult (dark green) and refined permissive upper bound (light green) models. (d) All MFs. Adult null model, p-value (real < null) = 0.5080, Cohen's d = -0.053; Refined permissive upper-bound null model, p-value (real < null) = 0.0000, Cohen's d = -4.921. (e) only VGluT1+ MFs. Adult null model, p-value (real < null) = 0.3530, Cohen's d = -0.469; Refined permissive upper-bound null model, p-value (real < null) = 0.0000, Cohen's d = -3.294. (f) only VGluT1– MFs. Adult null model, p-value (real < null) = 0.3810, Cohen's d = -0.474; Refined permissive upper-bound null model, p-value (real < null) = 0.0060, Cohen's d = -2.257.

**
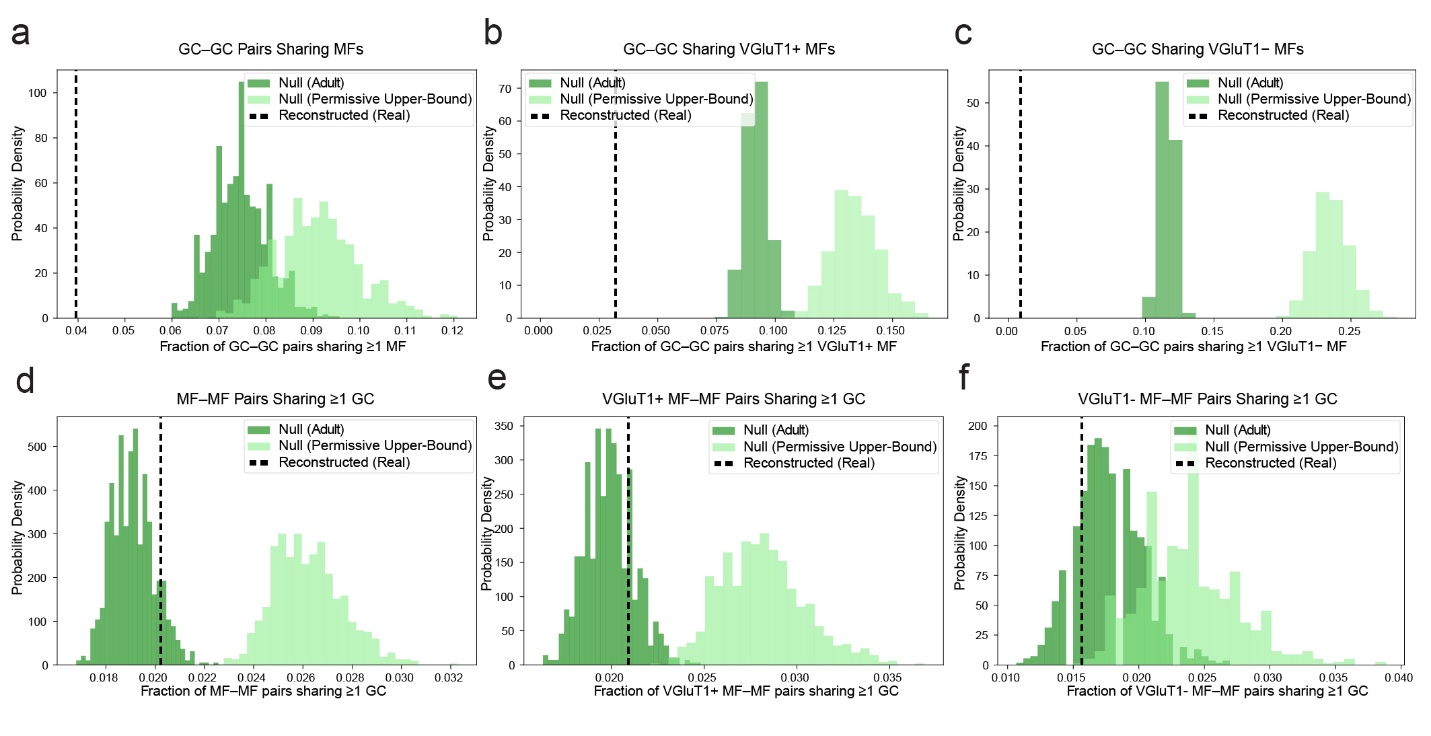
**

**Sup. Figure 4. Results from the adult model and the permissive upper-bound model after excluding granule cell near the upper boundary of the reconstructed volume.**

(a–c) Probability density distributions of the fraction of GC–GC pairs sharing ≥1 MF. Real data (black dashed line) are compared with null distributions from the adult (dark green) and permissive upper-bound (light green) models. (a) All MFs. Adult null model, p-value (real < null) = 0.0000, Cohen's d = -6.012; Permissive upper-bound null model, p-value (real < null) = 0.0000, Cohen's d = -5.888. (b) only VGluT1+ MFs. Adult null model, p-value (real < null) = 0.0000, Cohen's d = -12.471; Permissive upper-bound null model, p-value (real < null) = 0.0000, Cohen's d = -9.981. (c) only VGluT1– MFs. Adult null model, p-value (real < null) = 0.0000, Cohen's d = -22.239; Permissive upper-bound null model, p-value (real < null) = 0.0000, Cohen's d = -17.612.

(d-f) Probability density distributions of the fraction of MF–MF pairs sharing ≥1 GC. Real data (black dashed line) are compared with null distributions from the adult (dark green) and permissive upper bound (light green) models. (d) All MFs. Adult null model, p-value (real < null) = 0.8970, Cohen's d = 1.322; Permissive upper-bound null model, p-value (real < null) = 0.0000, Cohen's d = -4.263. (e) only VGluT1+ MFs. Adult null model, p-value (real < null) = 0.8040, Cohen's d = 0.770; Permissive upper-bound null model, p-value (real < null) = 0.0000, Cohen's d = -3.192. (f) only VGluT1– MFs. Adult null model, p-value (real < null) = 0.2480, Cohen's d = -0.812; Permissive upper-bound null model, p-value (real < null) = 0.0010, Cohen's d = -2.225.

**
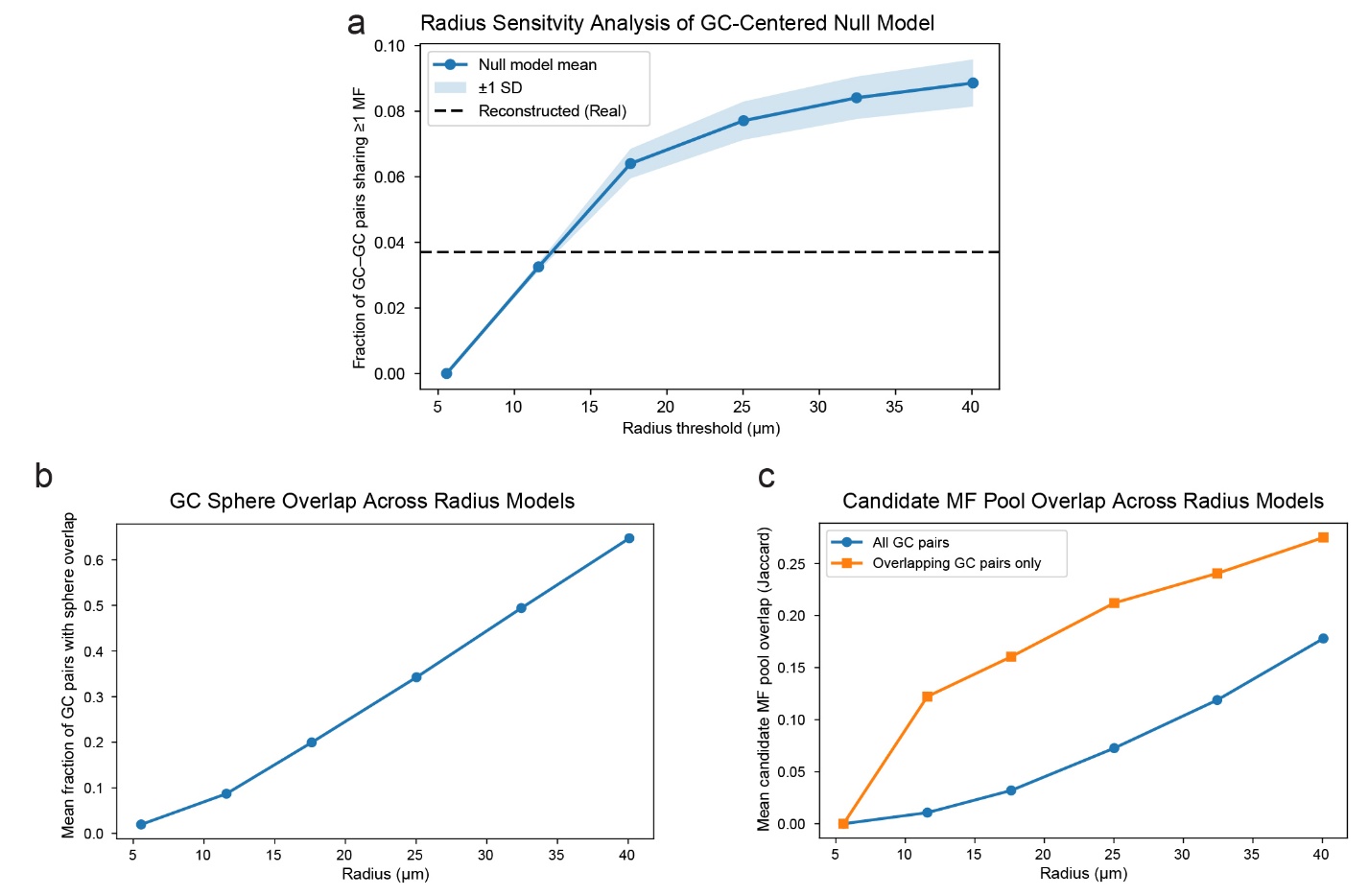
**

**Sup. Figure 5. Radius sensitivity analysis and geometry baseline of the spatial sampling null models.**

(a) Radius sensitivity analysis of the GC-centered null model. The expected fraction of GC pairs sharing ≥1 MF was evaluated across six sampling radii spanning the empirical range of dendritic reach. The null prediction (mean ± SD) increases monotonically with radius, whereas the empirical value from the reconstructed dataset (dashed line) remains constant. (b) Geometric overlap between granule cell sampling spheres. The mean fraction of GC pairs whose sampling spheres overlap increases with sampling radius. (c) Overlap of candidate MF terminal pools available to GC pairs across radii, quantified by the Jaccard index. Overlap is shown for all GC pairs (blue line) and for geometrically overlapping GC pairs only (orange line).

**
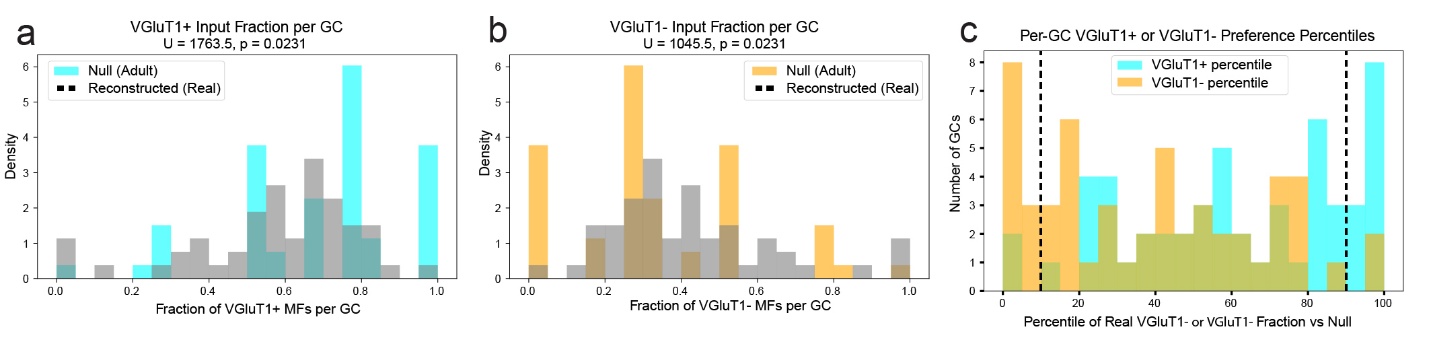
**

**Sup. Figure 6. Fraction of VGluT1-positive and VGluT1-negative terminals contacted by each granule cell with corresponding percentile ranks relative to random sampling, computed from a single Monte Carlo simulation.**

(a, b) Fractions of VGluT1+ (g) or VGluT1– (h) MF inputs per GC in the real dataset (cyan/orange) compared to the adult null model (gray). Mann–Whitney U test, VGluT1+: U = 1763.5, p = 0.0231; VGluT1–: U = 1045.5, p = 0.0231.

(c) Percentile ranks of each GC’s VGluT1+ or VGluT1– input fraction relative to the adult null model. Dashed lines indicate the 10th percentile and the 90th percentile.

**
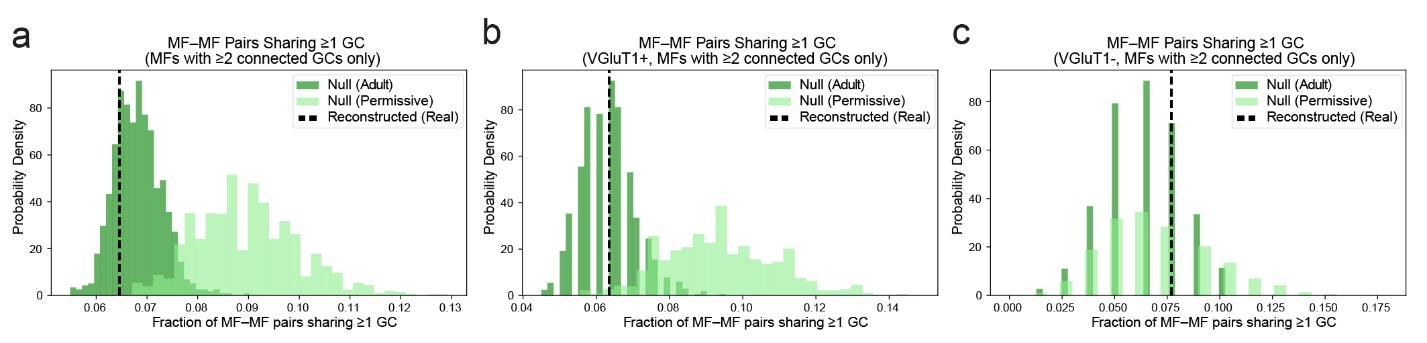
**

**Sup. Figure 7. Results from the adult model and the permissive upper-bound model on mossy fiber terminals connected to two or more granule cells.**

(a–c) Probability density distributions of the fraction of MF–MF pairs sharing ≥1 GC. Real data (black dashed line) are compared with null distributions from the adult (dark green) and permissive upper bound (light green) models. (a) All MFs connected to two or more granule cells (41 MFs in total). Adult null model, p-value (real < null) = 0.3040, Cohen's d = -0.702; Permissive upper-bound null model, p-value (real < null) = 0.0000, Cohen's d = -2.502. (b) only VGluT1+ MFs connected to two or more granule cells (28 MFs in total). Adult null model, p-value (real < null) = 0.6190, Cohen's d = 0.052; Permissive upper-bound null model, p-value (real < null) = 0.0170, Cohen's d = -1.973. (c) only VGluT1– MFs connected to two or more granule cells (13 MFs in total). Adult null model, p-value (real < null) = 0.8660, Cohen's d = 0.755; Permissive upper-bound null model, p-value (real < null) = 0.7190, Cohen's d = 0.259.

**Sup. Table. 1 Each Granule Cell’s Average Dendritic Reach**

| GC_ID | Avg_GC_MF_Distance_um |
| --- | --- |
| 0 | 14.03431 |
| 1 | 13.91273 |
| 14 | 20.20393 |
| 5 | 17.63436 |
| 2 | 15.28792 |
| 13 | 17.41242 |
| 6 | 19.1884 |
| 3 | 15.42373 |
| 52 | 19.19106 |
| 4 | 18.10126 |
| 12 | 15.65404 |
| 7 | 14.80607 |
| 8 | 20.17527 |
| 9 | 20.8857 |
| 44 | 18.42368 |
| 29 | 18.68063 |
| 41 | 21.11365 |
| 10 | 19.53384 |
| 27 | 17.74132 |
| 11 | 17.60073 |
| 23 | 16.10864 |
| 24 | 19.52543 |
| 15 | 13.65888 |
| 20 | 17.39071 |
| 21 | 19.94994 |
| 16 | 22.53306 |
| 18 | 13.23784 |
| 17 | 12.69762 |
| 19 | 14.83283 |
| 22 | 19.17102 |
| 31 | 22.26223 |
| 32 | 16.40423 |
| 25 | 16.32325 |
| 26 | 19.73753 |
| 28 | 19.55951 |
| 45 | 16.55877 |
| 30 | 21.30647 |
| 42 | 26.61571 |
| 33 | 15.55757 |
| 38 | 18.53717 |
| 34 | 15.90922 |
| 35 | 14.82666 |
| 36 | 12.21775 |
| 37 | 17.12128 |
| 39 | 18.0756 |
| 40 | 19.16219 |
| 43 | 16.6299 |
| 51 | 14.08654 |
| 46 | 16.93076 |
| 47 | 17.838 |
| 50 | 17.31924 |
| 48 | 20.47394 |
| 49 | 14.68041 |

**Sup. Table. 2 Each Granule Cell’s Maximum Dendritic Reach**

| GC_ID | Max_GC_MF_Distance_um |
| --- | --- |
| 0 | 20.97354 |
| 1 | 19.02233 |
| 14 | 25.79318 |
| 5 | 27.42147 |
| 2 | 18.42334 |
| 13 | 25.27892 |
| 6 | 28.47058 |
| 3 | 18.0756 |
| 52 | 22.43912 |
| 4 | 23.61468 |
| 12 | 23.62411 |
| 7 | 18.35987 |
| 8 | 31.15861 |
| 9 | 34.39213 |
| 44 | 27.72668 |
| 29 | 20.60123 |
| 41 | 32.8364 |
| 10 | 21.09243 |
| 27 | 25.07327 |
| 11 | 30.31794 |
| 23 | 22.75622 |
| 24 | 31.72236 |
| 15 | 15.54357 |
| 20 | 18.72427 |
| 21 | 28.47868 |
| 16 | 28.03982 |
| 18 | 18.46976 |
| 17 | 13.59692 |
| 19 | 18.06321 |
| 22 | 25.20496 |
| 31 | 40.0763 |
| 32 | 23.68594 |
| 25 | 28.71626 |
| 26 | 26.75963 |
| 28 | 24.37795 |
| 45 | 34.06286 |
| 30 | 29.1007 |
| 42 | 36.70663 |
| 33 | 27.71189 |
| 38 | 23.53461 |
| 34 | 30.49674 |
| 35 | 21.32158 |
| 36 | 14.72448 |
| 37 | 26.93054 |
| 39 | 23.06253 |
| 40 | 23.90999 |
| 43 | 22.2518 |
| 51 | 19.04997 |
| 46 | 21.41323 |
| 47 | 22.61296 |
| 50 | 23.02823 |
| 48 | 24.24645 |
| 49 | 19.82053 |
